# 

**Published:** 1851-09

**Authors:** 


### ARTICLE VI

### NOTICE TO READERS AND CORRESPONDENTS.

We have reccived original communications from Drs. Smick and McArthur, and will have for the next number of the Journal an interesting report of cases from the Illinois General Hospital of the Lake.

We have received the Transactions of the Medical Society of the State of New York for 1851; the Transactions of the New York Academy of Medicine for 1851; the Proceedings of the Medical Association of the State of Alabama for 1851; Prof. Jno. C. Warren's Address before the American Medical Association at Cincinnati, (from the author); Report of the Trial of Wm. R. Winton, M. D., (from the defendent); Minutes of the Proceedings of the Medical Society of the State of Georgia for 1851; Proceedings of the Twenty-Second Annual Meeting of the Medical Society of Tennessee, 1851; By-Laws of the Indiana Hospital for the Insane, 1851; an Address by Thos. E. Bond, A. M., M. D., to the Graduates of Washington University of Baltimore, 1851. We had written a notice of this Address, but it was unavoidably crowded out for want of room. An Address by Prof. A. Litton to the Graduates of the St. Louis University, 1851; the Valedictory Address of Prof. Downey to the Graduates of the Indiana Central Medical College, 1851: together with the Annual Announcements of the Medical Department of the University of Pennsylvania, of Jefferson Medical College, Starling Medical College, Medical Department of the University of Michigau, New York Medical College, Medical College of the State of South Carolina, University of New York, Western Reserve College, Kentucky School of Medicine, Medical Department of the St. Louis University, Medical College of Ohio, Hampden Sydney College, Richmond, Va., and cf the Indiaua Central Medical College. Also our usual list of exchanges.

---

### OBITUARY.

Died, at Elgin, Ill., Aug. 6th, of Consumption, Dr. Erastus Sanford, in the 31st year of his age.

The highest testimonials of his geod stahding in the profession and in society, have been furnished.

### NOTICE TO SUBSCRIBERS.

We are sorry that the Edition of No. 1 of the current volume of our Journal has been exhausted by the great number of new subscribers on our list, so that those who have lately sent in their names cannot be supplied. We will print a larger edition of the last half of the volume, so that those sending in hereafter will be permitted to begin in the middle of the volume. This, however, is to be the only variation from the rule that subscriptions must begin and close with the volumes as they are issued.

### RECEIPTS FOR THE JOURNAL FOR JULY & AUGUST.

ILLINOIS.

| | |
|---|---|
| Dr. S. Terwelliger, | $2 00 |
| D. E. Ellis, | 2 00 |
| D. W. Boyd, | 2 00 |
| N. Sapp, | 2 00 |
| A. Castle, | 5 00 |
| I. G. Harlan, | 1 00 |
| James Adair, | 1 00 |
| E. Thomas, | 2 00 |
| D. M. Cool, | 2 00 |
| Jesse Bennett, | 2 00 |
| F. C. Hageman, | 2 00 |
| W. B. Hart, | 2 00 |
| J. H. Wilkinson, | 2 00 |
| A. S. Haskell, | 2 00 |
| J. G. Tilden, | 2 00 |
| F. B. Ives, | 2 00 |
| J. J. Lescher, | 2 00 |
| G. W. Southwick, | 2 00 |
| W. Smith, | 4 00 |
| H. C. Donaldson, | 2 00 |
| F. B. Haller, | 2 00 |
| T. Leeds, | 2 00 |
| B. Comstock, | 6 00 |
| Witty & Irwin, | 2 00 |
| Allen, | 4 00 |
| James Leighton, | 2 00 |
| T. T. Brooks, | 6 00 |
| I. S. Evans, | 2 00 |
| George Allen, | 2 00 |
| H. L. Strong, | 2 00 |
| J. B. Fox, | 2 00 |
| A. G. Porter, | 2 00 |
| A. Ballow, | 2 00 |
| G. A. Gorham, | 3 00 |
| L. F. Avery, | 2 00 |
| J. F. Weeks, | 2 00 |
| E. Winslow, | 2 00 |
| G. Smith, | 4 00 |
| D. B. Jewell, | 4 00 |
| I. S. Ives, | 2 00 |
| D. K. Town, | 4 00 |
| F. Bry, | 2 00 |
| C. Wheeler, | 2 00 |
| Kimball Favour, | 2 00 |
| J. Burlingame, | 1 00 |
| E. J. Williams, | 2 00 |
| W. H. Crandal, | 4 00 |
| W. A. Little, | 1 00 |
| —— Babbitt, | 3 00 |
| Little & Bonney, | 2 00 |
| J. M. Russell, | 2 00 |
| Dr. H. Newhall, | $2 00 |
| —— Spillman, | 1 00 |
| C. M. Smith, | 1 50 |
| E. C. Ellett, | 2 00 |
| J. A. Halderman, | 2 00 |
| W. B. Brink, | 2 00 |
| M. A. Cooper, | 2 00 |
| J. B. Samuel, | 2 00 |
| J. R. Boyce, | 2 00 |
| J. Blount, | 4 00 |
| J. E. McGirr, | 2 00 |

INDIANA.

| | |
|---|---|
| Dr. Charles Brackett, | $2 00 |
| R. D. Manzy, | 4 00 |
| W. Bassett, | 2 00 |
| C. S. Perkins, | 2 00 |
| B. S. Noble, | 2 00 |
| V. P. Kennedy, | 2 00 |
| D. Hutchinson, | 2 00 |
| Thos. A. Graham, | 1 00 |
| Hiram Duncan, | 2 00 |
| H. P. Howes, | 8 00 |

WISCONSIN.

| | |
|---|---|
| Dr. G. S. Maxson, | $2 00 |
| C. D. Schenech, | 2 00 |
| Storrs Hall, | 2 00 |
| John Ingalls, | 2 00 |
| John Currie, | 3 00 |
| Reuben Hall, | 2 00 |
| A. S. Palmer, | 2 00 |
| —— Chamberlain, | 2 90 |
| J. A. Preston, | 2 00 |
| E. P. Wood, | 2 00 |
| —— Steel, | 2 00 |
| C. C. Terrell, | 2 00 |

MICHIGAN.

| | |
|---|---|
| Dr. P. D. McArthur, | $2 00 |
| J. G. Cornell, | 4 00 |
| D. Fitzgerald, | 2 00 |

OHIO.

| | |
|---|---|
| Dr. Orange Fisher, | $1 00 |

MASSACHUSETTS.

| | |
|---|---|
| Dr. Walter Channing, | $6 00 |

ARKANSAS.

| | |
|---|---|
| Wm. Wilson, Esq., | $2 00 |

